# Arthroscopic Posterior Glenoid Osteotomy

**DOI:** 10.1016/j.eats.2023.09.003

**Published:** 2023-12-18

**Authors:** Abdul-ilah Hachem, Jhonattan Pereira, Xavi Rius, Alex Campagnoli

**Affiliations:** a Department of Orthopedic Surgery, Bellvitge University Hospital (L’Hospitalet de Llobregat), Barcelona, Spain; b Shoulder Unit, Bellvitge University Hospital, and Associated Professor of the University of Barcelona, Spain; c Master Fellowship in Shoulder Surgery at University of Barcelona (L’Hospitalet de Llobregat), Barcelona, Spain; d Arthrex Gmbh, Munich, Germany

## Abstract

Management of posterior shoulder instability in patients with excessive glenoid retroversion can be challenging. However, a corrective posterior glenoid osteotomy is an option. Although various open techniques are available, minimally invasive and arthroscopy surgery are the most advantageous. This study describes the feasibility and safety of an arthroscopic posterior open wedge glenoid osteotomy using an autologous scapular spine graft along with additional posterior capsulolabral complex reattachment. This procedure is a viable option for patients with symptomatic posterior shoulder instability.

Posterior shoulder instability (PSI) represents around 2% to 10% of all instabilities.^1^ Classification of PSI is challenging and traditionally has been described after acute trauma or atraumatic or repetitive microtrauma.^2^ Atraumatic PSI is most common and is often associated with generalized ligamentous laxity and increased glenoid retroversion.^3^ The regular glenoid version was –4° (range, –11° to 5°) on computed tomography scans in Graichen et al.^4^ This is consistent with other glenoid versions analyzed of 4° to –7° of retroversion.^5^^,^^6^ Excessive retroversion of the glenoid causes eccentric loading of the glenohumeral joint and can lead to instability, progressive arthritis, functional impairment, and posterior subluxation.^7^

Glenoid dysplasia with the osseous posterior rim deficiency increases retroversion and hyperplasia of the posterior labrum.^8^ This can be associated with recurrent posterior subluxation of the humeral head (PSH).^9^

Symptoms of PSI present a challenge in diagnosis and treatment as they are often vague and nonspecific.^2^ Conservative treatment improves symptoms, such as strengthening the rotator cuff muscles and controlling proprioception.^10^ Surgical treatment involves soft tissue, osseous procedures, or a combination.^6^ Hurley et al.^11^ found that patients with symptomatic posterior instability and glenoid retroversion of >9° experienced higher recurrence rates after soft tissue procedures. Biomechanical studies have shown glenoid retroversion of >10°; soft tissue repair may not be sufficient to treat PSI.^12^ Osseous procedures are categorized as either glenoid augmentation (bone graft) or glenoid reorientation (osteotomy).^6^

Scott^13^ first described corrective posterior glenoid osteotomy (PGO) in 3 cases with chronic posterior dislocation. Several studies show that posterior open wedge glenoid osteotomy can successfully treat excessive retroversion and insufficient concavity in atraumatic posterior instability.^8^^,^^14^^,^^15^ There is no clear indication for PGO, but it is a treatment option for young patients with excessive glenoid retroversion with recurrent symptomatic instability.^3^ Osteotomy of the glenoid is a demanding technique, and consequently, complications can be substantial, including intra-articular fracture, graft extrusion, overcorrection, or loss correction with subsequent development of osteoarthritis.^16^ Recent advances in arthroscopic techniques allow arthroscopic posterior bone grafts to provide a congruent extension of the articular surface.^6^^,^^17^

Among the improvements in the instrumentation, this procedure has become a viable option in treating glenoid retroversion. This is a universally recognized challenging procedure. An absolute understanding of the lateral decubitus position in arthroscopic anatomy helps improve the learning curve of performing this technique. In this study, we present the feasibility of showing a safe, reproducible, and practical approach using a specific instrument for an arthroscopic PGO as a treatment option for patients with excessive retroversion associated with glenoid dysplasia.

The advantages and disadvantages of this technique are discussed in Table 1.

**Table 1:** Advantages and Disadvantages

Advantages
• A minimally invasive technique under scope visualization.
• Better control in positioning the drill guide parallel to the joint line.
• It can be used for autograft.
• Stable and metal-free procedure impacting the bone graft.
• Preserves joint and the capsulolabral complex.
• No intraoperative x-ray is needed.
• Feasible, strong, safe, and reproducible technique.
Disadvantages
• The risk of glenoid fracture.
• Risk of the suprascapular nerve lesion working to medial to the posterior glenoid rim.

## Preoperative Planning

Assessment of the angle of glenoid retroversion is mandatory for the comprehensive management of PSI. The Friedman method is commonly the most helpful tool to determine retroversion, and computed tomography (CT) scans allow accurate measurements.^9^ To determine the glenoid version first, draw a line from the glenoid fossa's center point to the scapula image's medial extremity. Next, draw a perpendicular line (forming 90°) to the previous axial line. Finally, draw a line between the anterior and posterior margins of the glenoid. The angle formed by these lines is the glenoid version. Magnetic resonance imaging (MRI) provides adequate information about the posterior capsulolabral complex and CT-guided surgical decision-making for patients with PSI. The indication for arthroscopic PGO is in patients with symptomatic recurrent posterior shoulder instability with glenoid retroversion greater than 25°.

## Surgical Technique

The surgical technique is demonstrated in Video 1.

### Patient Position

**Fig 1:**
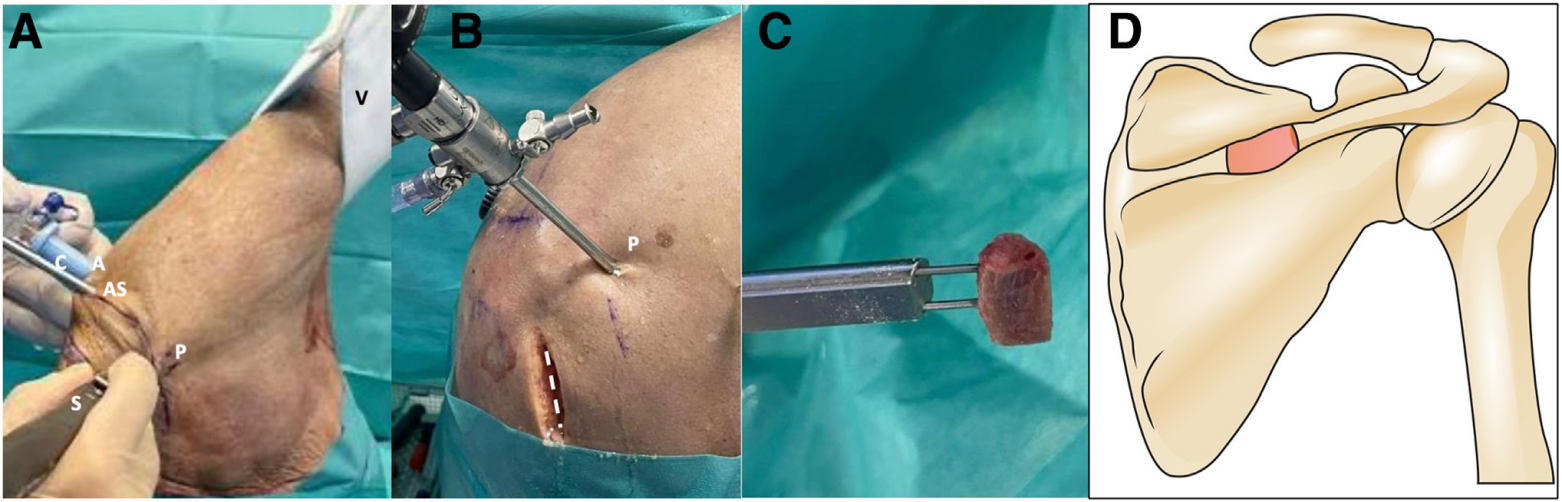
Right shoulder, lateral decubitus position, external view. (A) Arthroscopic scope was inserted through the anterosuperolateral portal, Shaver handpiece through the posterior standard portal, and the blue cannula (C) through the anterior rotator interval portal. (B) The dotted marks indicate the horizontal approach over the midpoint of the scapula spine for bone autograft harvesting. (C) Triangular-shaped tricortical bone scapular spine autograft. (D) The dorsal view of the right scapula depicts the specific area from where the graft is harvested. (A, anterior portal; AS, anterosuperolateral portal; C, cannula; P, posterior portal; S, Shaver handpiece; V, lateral vertical strap.)

The patient is positioned in the lateral decubitus position with a 30° posterior obliquity aligning the glenoid parallel to the floor. The arm is placed in a traction foam sleeve (3-point Shoulder Distraction System; Arthrex) (Fig 1A).

### Scapular Spine Autograft Preparation

The midpoint width of the scapular spine is identified, and a horizontal incision measuring 3 to 4 cm is made, located 5 cm laterally to the medial scapular border. The posterior deltoid insertion and trapezius fascia are dissected to expose the scapular spine^18^ (Fig 1B). After harvesting the triangular-shaped tricortical bone graft, typically measuring 20 × 10 × 8 mm, a saw and an osteotome are used to close the fascia and the skin (Fig 1C, D).

### Arthroscopic Glenoid Preparation

**Fig 2:**
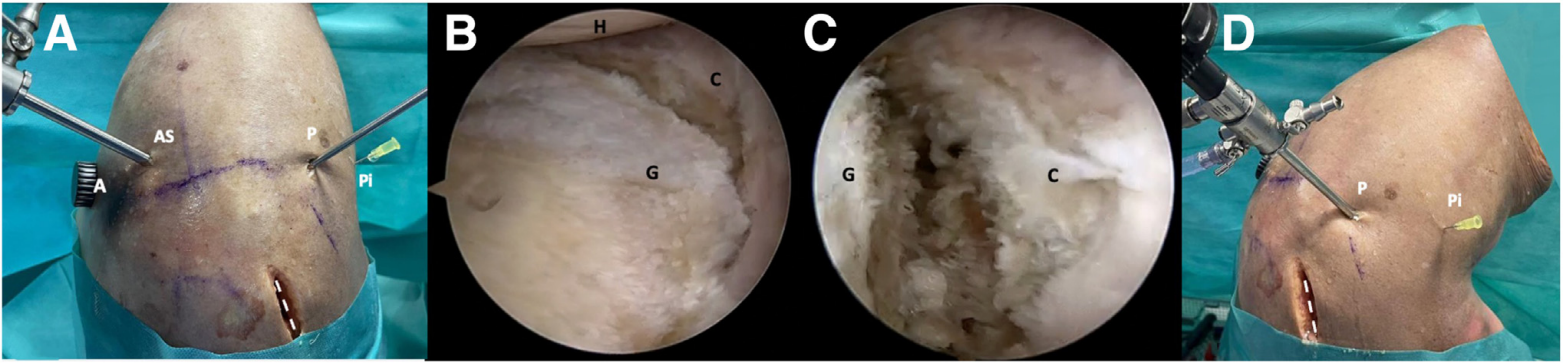
Right shoulder, lateral decubitus position. (A) Extra-articular view shows the routine portals: posterior, anterior, anterosuperolateral (ASL), and an accessory posteroinferior portal. A switching stick is inserted through the standard posterior portal. (B, C) Arthroscopic view from the ASL portal. Notice the complete detachment of the posterior capsulolabral complex and thorough debridement of the posterior glenoid rim and wall. (D) The needle is introduced to establish the appropriate level for the posteroinferior working osteotomy portal. The dotted line marks the scapula spine approach. (A, anterior portal; AS, anterosuperolateral portal; C, capsulolabral complex; G, glenoid; H, humerus; P, posterior portal; Pi, posteroinferior portal.)

The affected shoulder is maintained in 20° to 30° of abduction, applying 3 to 4 kg of traction. The lateral vertical strap of the foam sleeve traction system is set up to enhance access to the glenohumeral joint and the axillary pouch (Fig 1A). To successfully perform this procedure, it is necessary to use the following routine portals: posterior, anterior, anterosuperolateral (ASL), and an accessory posteroinferior portal. The procedure starts with the scope through the standard posterior portal (Fig 2A), followed by the anteroinferior portal through the rotator interval. A diagnostic arthroscopy is performed to assess the glenohumeral joint, examining all structures with particular attention to the glenoid cartilage surface, posterior capsulolabral complex, and the presence of a humeral reverse Hill-Sachs lesion. Afterward, the scope is switched to the ASL portal located directly behind the biceps tendon.

To perform the osteotomy accurately, it is necessary to release the posterior capsulolabral complex (CC). It is crucial to detach the posterior labrum from the long head of the biceps tendon insertion at 11- to 10:30-o’clock and elevate it from the posterior glenoid rim down to the 6-o’clock position. Using a soft tissue elevator and a radiofrequency device, we ensure complete visibility and exposure of the posterior scapula wall (Fig 2B, C). To ensure proper positioning of the labrum and maximize the expansion of the posterior articular pouch, a percutaneous monofilament suture encircles both the CC and labrum laterally and medially.

A percutaneous needle should establish the correct posteroinferior working osteotomy portal level. Ensuring the needle is parallel to the glenoid surface and positioned at the midpoint of the superoinferior posterior glenoid rim is essential (Fig 2D).

**Fig 3:**
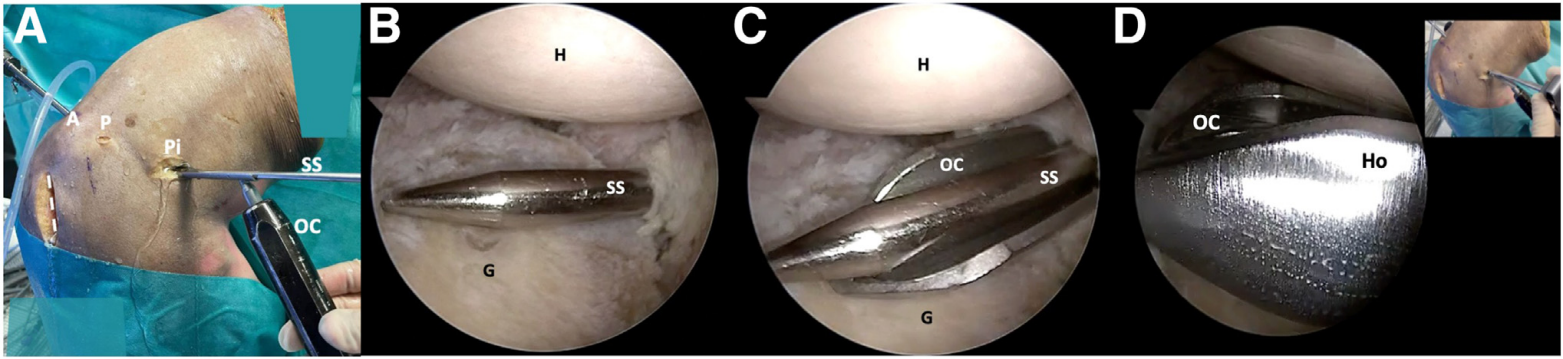
Right shoulder, lateral decubitus position. (A) Extra-articular view. The open metallic cannula is introduced underneath the switching stick through the accessory posteroinferior portal. (B, C) Arthroscopic view from the AS portal depicts the switching stick and the open metallic cannula. (D) The rod is exchanged with the drill guide hook. The dotted line marks the scapula spine approach. (AS, anterosuperolateral portal; G, glenoid; H, humerus; Ho, drill guide hook; P, posterior portal; Pi, posteroinferior portal; OC, open metallic cannula; SS, switching stick.)

**Fig 4:**
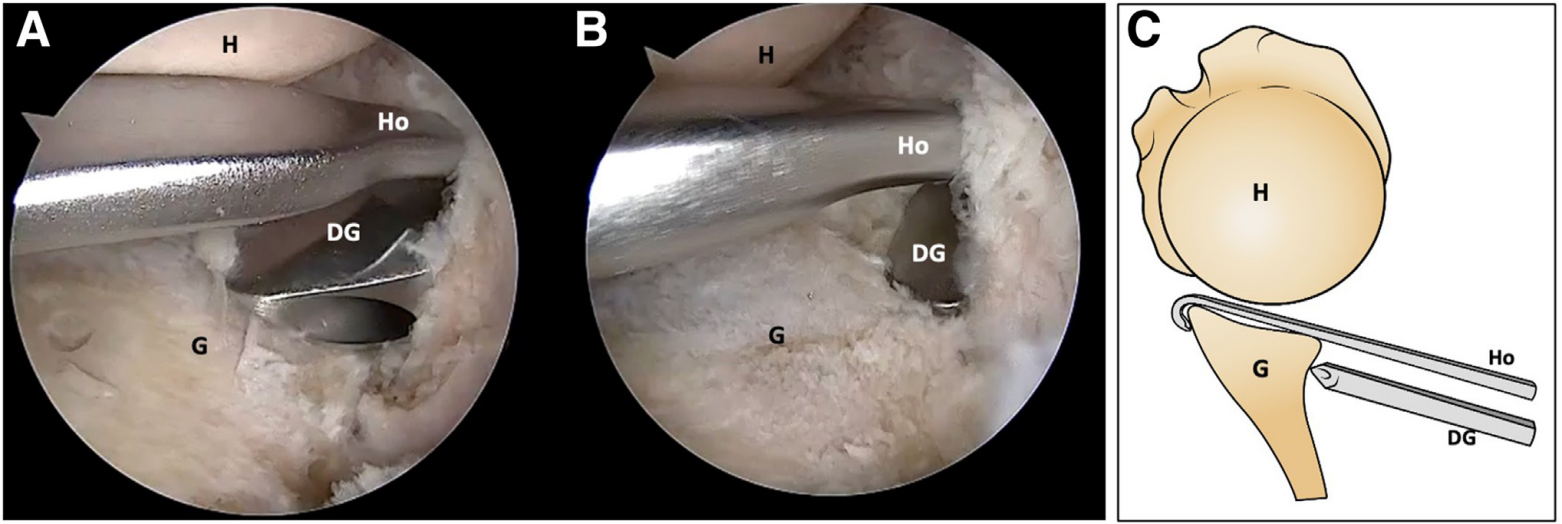
Right shoulder, lateral decubitus position. (A, B) Arthroscopic view. The drill guide sleeve assembled to the handle of the drill guide hook is introduced from the posteroinferior working portal. (C) Schematic representation illustrates the specific hook and drill guide. (DG, drill guide; G, glenoid; H, humerus; Ho, drill guide hook.)

A switching stick is inserted through the posteroinferior portal into the joint, positioning it from posterior to anterior at the level of the glenoid surface. It is centered at the midpoint distance from superior to inferior of the posterior glenoid rim. An open metallic cannula (Arthrex) is introduced over or underneath the switching stick. Subsequently, the stick is exchanged with the hook of the drill introducing it under the guidance of the open metallic cannula (Fig 3). To provide sufficient access, the incision of the posteroinferior portal must be vertically enlarged along the posterior glenoid rim (Fig 3A). To facilitate the introduction of a specific guide and create a working space for osteotomy devices under the laterally separated capsulolabral complex, it is recommended to use Mayo scissors to split the fibers of the infraspinatus muscle. Subsequently, the drill guide is assembled to the handle of the drill guide hook (Fig 4).

### Glenoid Osteotomy

Advanced support instruments are in Table 2.

**Table 2:** Advanced Support Instruments

When using the drill guide sleeve, it is essential to make sure that the straight aiming guide is flush with the surface of the glenoid cartilage and especially the posterior and anterior rims.

**Fig 5:**
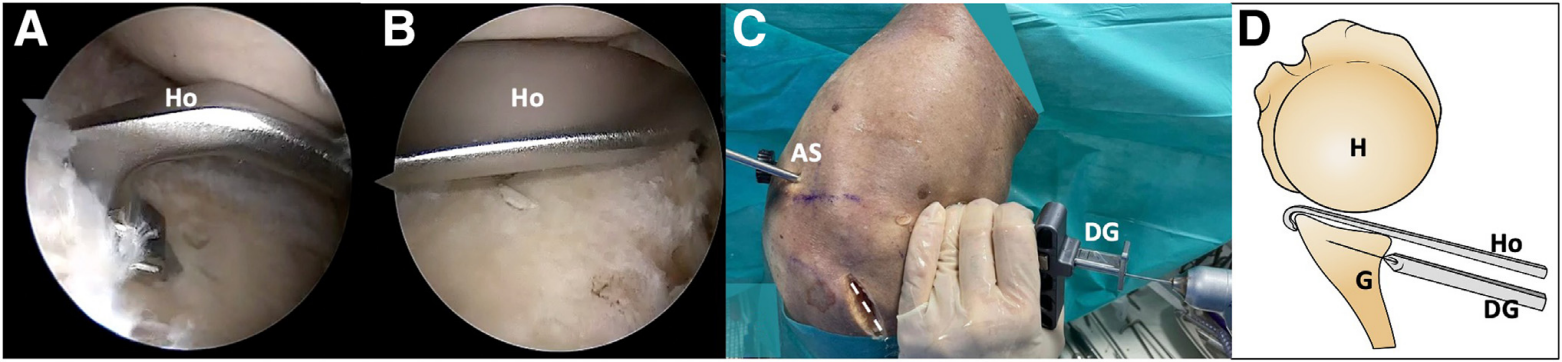
Right shoulder, lateral decubitus position. Drill 2 k-wires through the specific drill guide. (A, B) Drill hook is introduced from the posteroinferior working portal. (C) External view. (D) Schematic representation; the k-wires through the specific drill guide stop short of breaking through the anterior cortex. The dotted line marks the scapula spine approach. (AS, arthroscopy scope; G, glenoid; H, humerus; DG, specific drill guide; Ho, drill guide hook.)

Choose the 7-mm tunnel offset and insert the drill guide sleeve with the 7-mm side facing the hook’s shaft. The sleeve has laser markings indicating the intraosseous distance between the posterior and anterior scapular bone. These markings are calculated from the tip of the sleeve to the drill exit points. To prevent reaching the anterior cortex, it is essential to use 2 k-wires with less than a 3-mm diameter and 1 cm apart with label markings to calculate the proper length accurately. By following these instructions carefully, you can ensure that the drill tunnels using the k-wires are shorter than the measured distance of the glenoid obtained with the guide. This step is crucial for accurate and precise drilling within the glenoid to avoid breaking through the anterior cortex (Fig 5).

**Fig 6:**
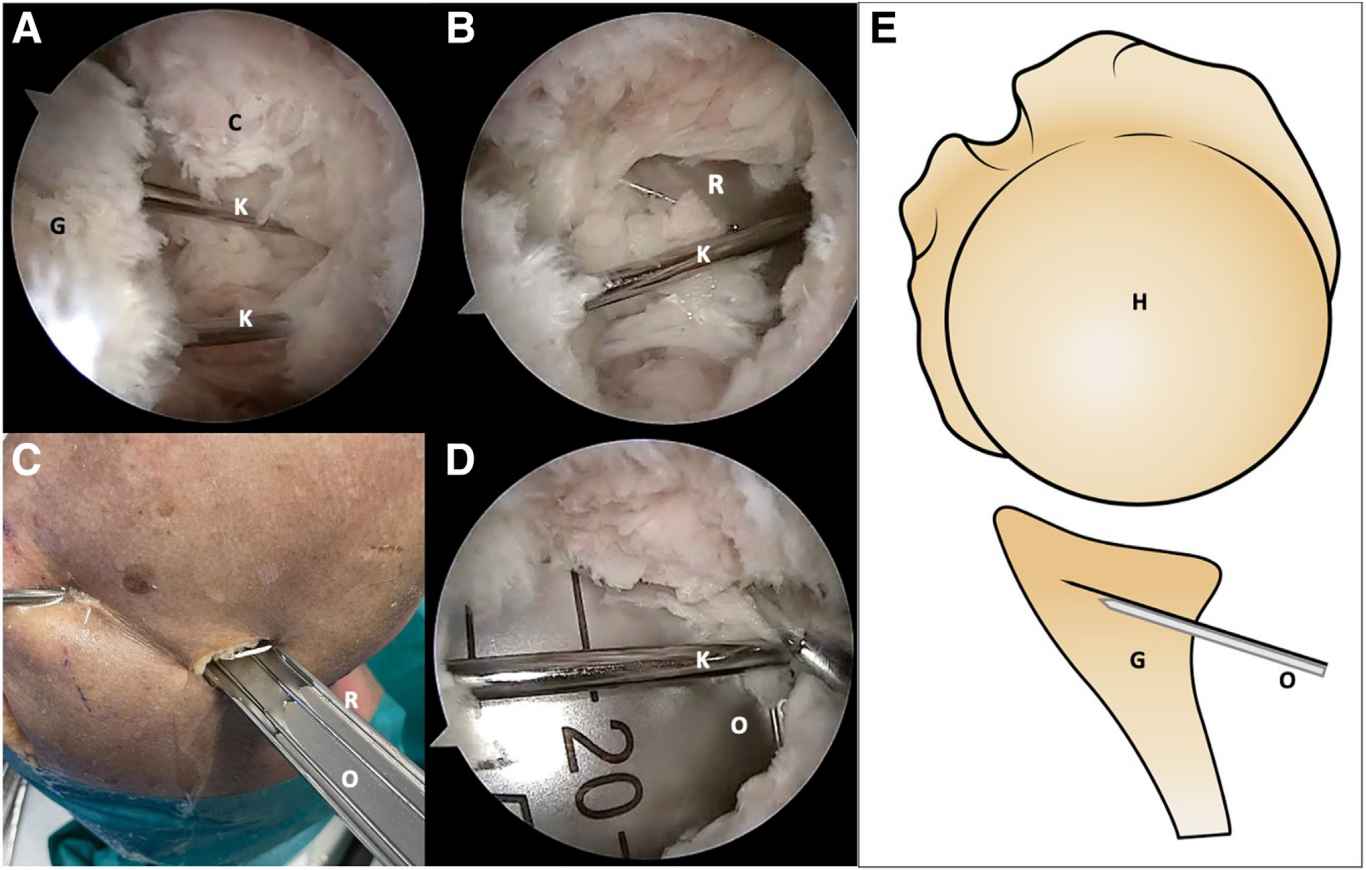
Right shoulder, lateral decubitus position. (A, B) Anterosuperolateral scope view. The k-wires are exposed, and the forked retractor is posteriorly introduced. (C) External view. A forked retractor with rails is inserted beneath the k-wires. (D) The osteotome is introduced over the retractor and in between the k-wires, impacting into the bone. (E) Schematic representation. Notice the osteotome under the k-wires. (C, capsulolabral complex; G, glenoid; H, humerus; K, k-wires; O, osteotome; R, forked retractor.)

Once the drill guide has been removed, the k-wires are exposed posteriorly. Use direct scope visualization to properly insert both the forked retractor with a 15-mm width and raised rails, as well as the blunt retractor gently beneath the k-wires. The rails on either side of the retractor will guide the osteotome and prevent harm to the infraspinatus fibers during insertion. Make sure to position them against the posterior glenoid wall. After the blunt retractor is removed, there will be enough space for the laser-marked osteotome to be used. The forked retractor is then partially removed, and the osteotome is introduced and carefully impacted into the bone until the 15-mm laser mark is reached (Fig 6).

**Fig 7:**
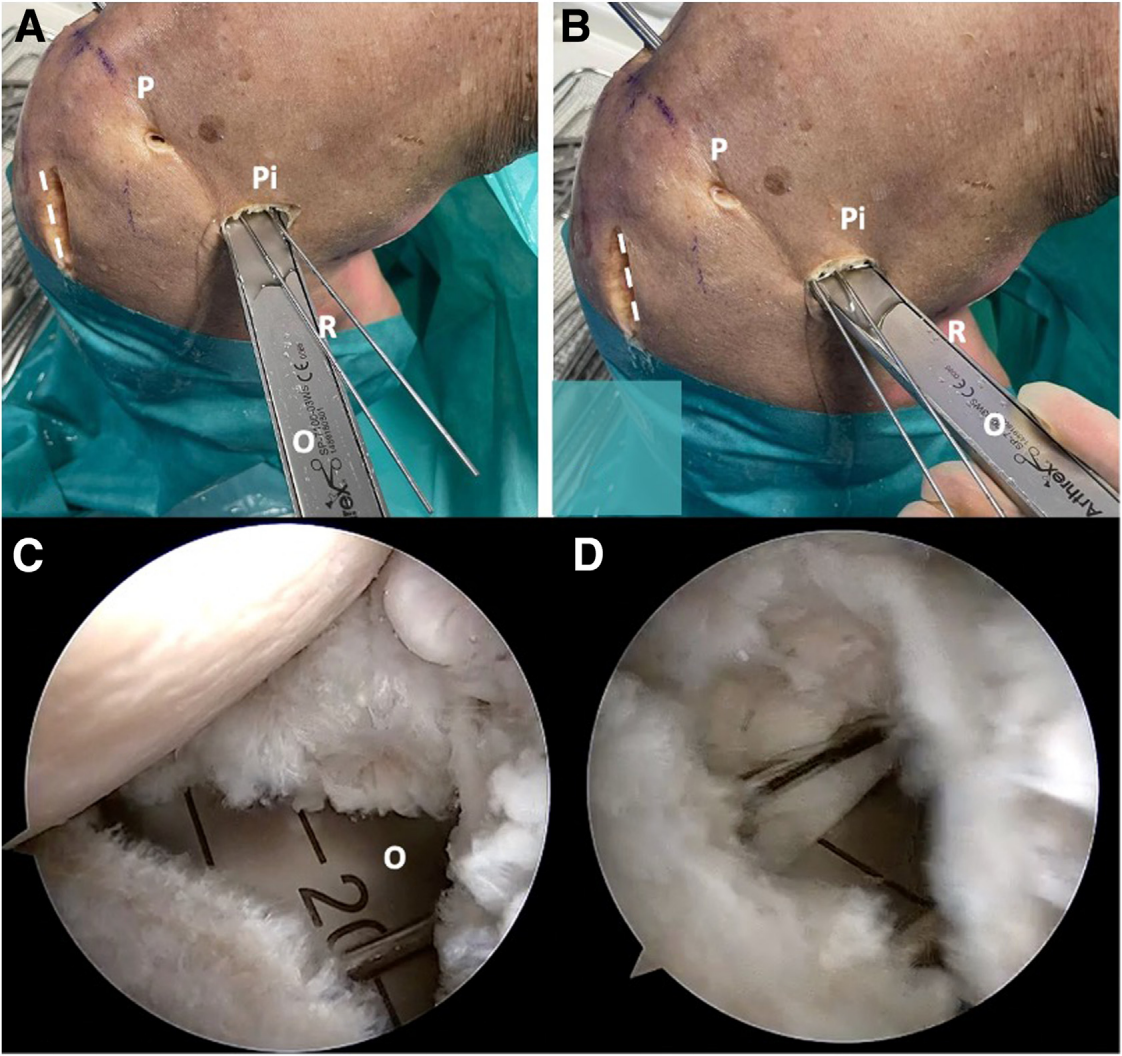
Right shoulder, lateral decubitus position. Change the orientation of the cut inferiorly and superiorly to complete the osteotomy. Posterior external view: (A) inferior orientation and (B) superior orientation. Arthroscopic view: (C) notice the osteotome with inferior and (D) with superior direction. The dotted line marks the scapula spine approach. (O, osteotome; P, posterior portal; Pi, posteroinferior portal; R, forked retractor.)

**Fig 8:**
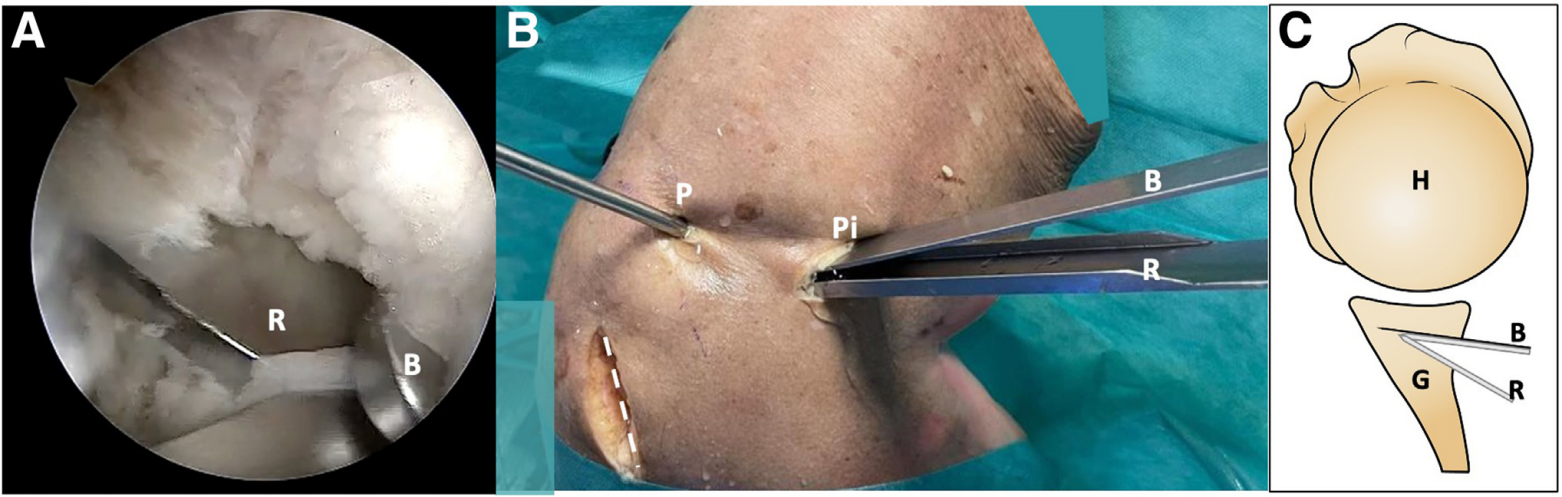
Right shoulder, lateral decubitus position. (A) Arthroscopic view. The forked retractor and the blunt retractor are introduced together into the posterior osteotomy. (B) External view. The forked and blunt retractors open the osteotomy wedge. (C) Schematic representation. The dotted line marks the scapula spine approach. (B, blunt retractor; G, glenoid; H, humerus; P, posterior portal; P, posterior portal; Pi, posteroinferior portal; R, forked retractor.)

Pull back the osteotome and change the orientation of the cutting tip inferiorly before impacting again to the desired 15-mm mark. Once again, retract the osteotome and change the direction of the cutting tip medially and superiorly to complete the glenoid osteotomy (Fig 7). Gently impact the osteotome into the bone and move it in all directions to achieve a complete opening osteotomy. Accurate placement and maintaining control during the osteotomy are crucial to avoid any fractures on the glenoid surface that could affect the overall success of the procedure. By using both the blunt or forked retractors and the osteotome tool, the open wedge osteotomy must be carefully opened under direct visualization of the glenoid cartilage (Fig 8).

### Graft Introduction and Fixation

**Fig 9:**
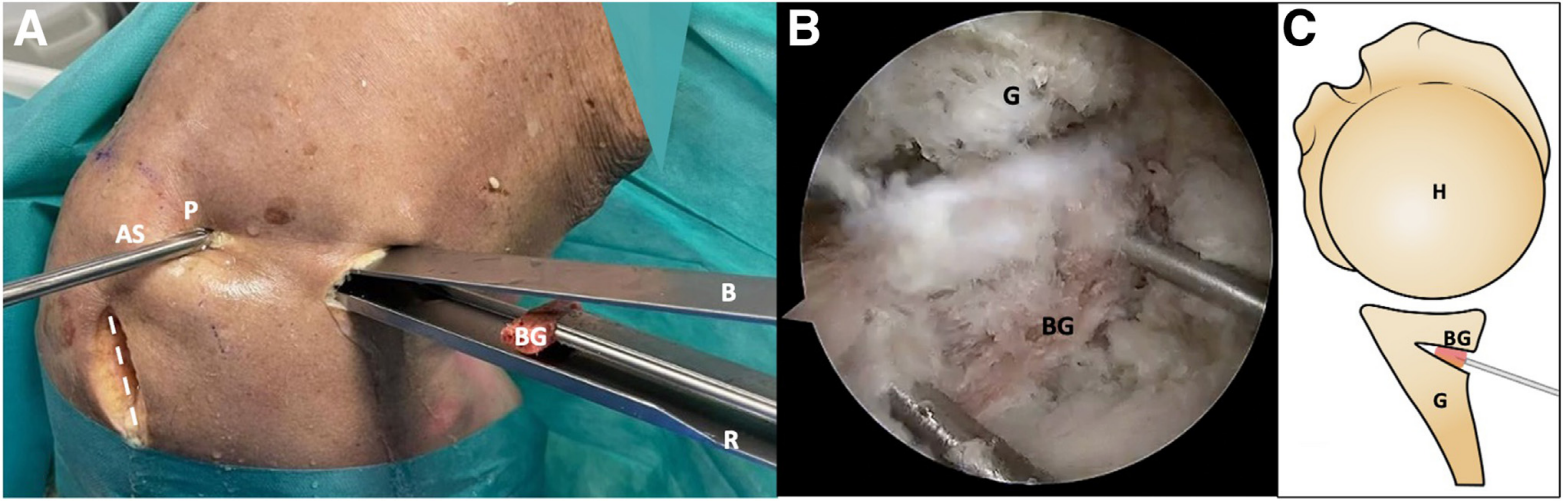
Right shoulder, lateral decubitus position. (A) The scapular spine autograft is introduced using 2 small k-wires, and then is slides over the retractor. (B) Arthroscopic view from the posterolateral portal. The graft is positioned using k-wires. (C) Schematic representation in axial view. The dotted line marks the scapula spine approach. (AS, arthroscopy scope; B, blunt retractor; BG, bone graft; G, glenoid; H, humerus; P, posterior portal; R, retractor.)

After harvesting the posterior scapular graft, it is fixed to the specific bone graft inserter using 2 small k-wires. The posterior scapular graft, held by the bone graft inserter, is introduced and carefully impacted into the previously created posterior glenoid opening osteotomy. Once the graft is securely and firmly seated, the small k-wires that secure it to the bone graft inserter are removed (Fig 9).

### Capsulolabral Repair

**Fig 10:**
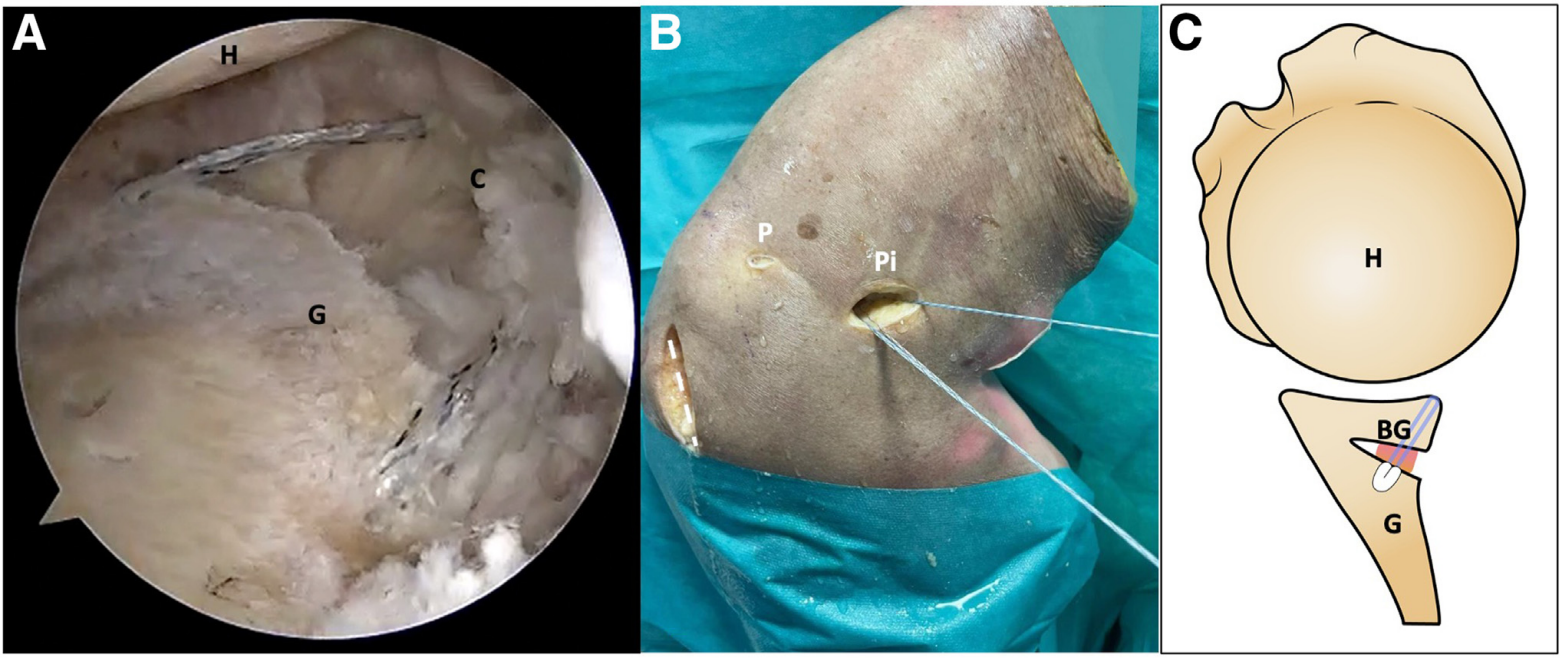
Right shoulder, lateral decubitus position. (A) Anterosuperolateral scope view portal. To reattach the capsulolabral complex to the posterior glenoid rim, 2 or 3 soft anchor implants are required to pass through the graft and reach the medial body of the scapula. (B) Posterior external view. Notice that 2 soft anchors have been inserted through the posteroinferior working portal. (C) Schematic representation. Axial view of the final procedure showing the use of an anchor for stabilizing both the osteotomy and graft. The dotted line marks the scapula spine approach. (BG, bone graft; C, capsulolabral complex; G, glenoid; H, humerus; P, posterior portal; Pi, posteroinferior portal.)

The capsulolabral complex is reattached to the edge of the posterior glenoid rim using 3 to 4 knotless 1.8-mm FiberTak (Arthrex) soft anchors with a nonfluted drill (Fig 10). To ensure graft stability in the osteotomy, at least 2 of the implants must pass through the graft with an appropriate angle to reach the medial body of the scapula. This helps provide additional fixation to the graft, ensuring its stability. An accessory percutaneous portal can be created to introduce anchors in the posteroinferior position, located laterally to the osteotomy working incision. This will enable a safe and correct angle of insertion.

**Fig 11:**
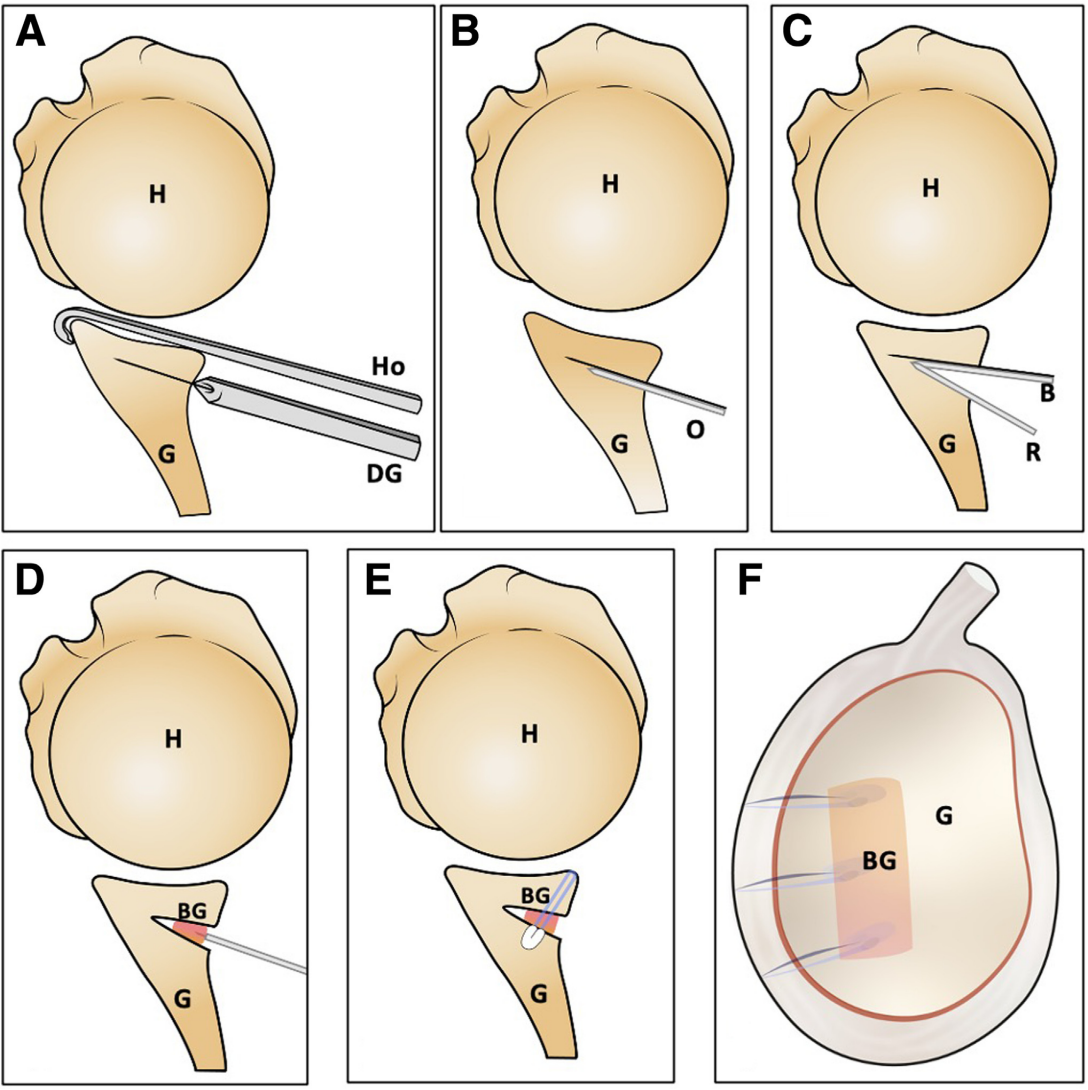
Schematic representation: the arthroscopic posterior glenoid osteotomy summary. (A) The specific drill guide is introduced through the posteroinferior working portal to drill 2 k-wires. (B) A forked retractor and osteotome are positioned between the k-wires. (C) The blunt retractor is positioned above the forked retractor to create and open the osteotomy. (D) The triangular scapular spine autograft is inserted and impacted while maintaining the open wedge osteotomy. (E) The capsulolabral complex is reattached to the posterior glenoid rim using implants that pass through the graft and reach the medial body of the scapula. (F) Final sagittal view. (B, blunt retractor; BG,bone graft; G, glenoid; H, humerus; Ho, drill guide hook; DG, specific drill guide; O, osteotome; R, forked retractor.)

The step-by-step schematic representation of the technique is presented in Figure 11. This technique's pearls, pitfalls, and limitations are presented in Table 3.

**Table 3:** Pearls, Pitfalls, and Limitations

Pearls
• Scapular graft harvesting is essential before arthroscopic surgery to avoid fluid swelling that can hinder the procedure.
• Detach the labrum at 11-o’clock behind the bicep insertion and release the capsulolabral complex thoroughly toward 6-o’clock.
• Align the needle with the glenoid surface for the perfect posteroinferior portal.
• For accuracy, position the hook drill guide correctly at the midpoint of the glenoid rim's backside.
• To insert the hook and drill sleeve, it is necessary to perform a broad split of the infraspinatus.
• Under direct scope view, the osteotome is inserted and moved upward through the center of the glenoid bone.
• Exercise caution using the retractor and osteotome when performing the opening wedge process.
• Use knotless soft anchors to reattach the labrum on the edge of the glenoid rim and ascertain the inclination angle to go through the graft and the scapular body.
Pitfalls
• Complete contact of the hook drill guide with the anterior and posterior glenoid rim warrants the accurate 7-mm offset of the k-wires.
• Enlarge the posteroinferior portal medially and parallel to the scapula spine to position the drill sleeve against the posterior glenoid wall.
• For joint line safety, do not insert the osteotome beyond the 20-mm mark in the glenoid.
Limitations
• A specific guide and instruments are needed.
• A demanding technique requiring a considerable learning curve.

## Discussion

Glenoid osteotomy is an uncommon surgery due to the limited indication, demanding surgical technique, and wide variety of results. Arthroscopic posterior glenoid osteotomy for patients with symptomatic instability and high glenoid retroversion is a feasible, safe, and acceptable treatment option with advanced arthroscopic shoulder surgeons.

There are no specific guidelines regarding the amount of retroversion necessary to determine if adding a posterior glenoid osteotomy or bone augmentation will result in successful outcomes.^12^ According to Hurley et al.,^11^ patients with posterior instability and glenoid retroversion exceeding 9° are more likely to experience recurrence after soft tissue procedures. Lacheta et al.^15^ considered the posterior instability with an additional glenoid retroversion over 10° to address the osteotomy.

In a recent study, Marcaccio et al.^5^ demonstrated that open wedge posterior osteotomy improved humeral translation resistance compared to isolated capsulolabral repair. In another biomechanical study, it was found that glenoid retroversion of more than 15° can result in the decentralization of the humeral head, which becomes a risk factor for failure after soft tissue repair procedures.^12^ Ernstbrunner et al.^19^ revealed that posterior opening osteotomy with the application of a J-shaped bone graft restored stability and normalized the glenohumeral contact pattern comparable to an intact glenoid. Furthermore, they have shown that the impacted graft without implant provides secure fixation. The same author conducted a study on 7 patients with atraumatic posterior shoulder instability with a minimum follow-up of 2 years. They used a posterior J-bone graft and reported that the median preoperative retroversion of 16° (ranging from 15° to 25°) was corrected to 0° postoperatively through successful bone union using a metal-free implant.^14^

Graichen et al.^4^ assessed 16 of 17 patients who underwent posterior glenoid osteotomy, clinically and radiologically, with an average follow-up of 5 years. According to the Constant and Rowe scores, the results indicate that 81% of the patients experienced good or excellent outcomes. Ortmaier et al.^7^ examined 10 shoulders from 8 patients. The Constant pain score improved from 6 to 11.1 points, and the overall score increased from 45 to 64. Moreover, the mean glenoid version was significantly modified from 11° to 5° postoperatively. In their study, Bessems and Vegter^20^ report the outcomes of 13 shoulders in 10 patients. The patients were followed up for an average of 9 years, ranging from 1 to 16 years. The results were excellent or good according to the Rowe score, and there were no instances of posterior dislocation recurrence. All patients reported being satisfied with the treatment and had a full range of motion. Pogorzelski et al.^21^ performed 6 posterior open wedge glenoid osteotomies, with an average follow-up of 26.8 months. They reported a mean postoperative retroversion of 11.2°, significantly lower than the presurgery measurement of 26.0°. However, it is noteworthy that 2 of the shoulders continued to show signs of persistent posterior instability.

Posterior glenoid osteotomy has an overall complication rate of 18.3%; the most significant complication, affecting 7.3% of patients, is the development of degenerative changes during clinical follow-up, followed by persistent instability.^3^ Other relevant complications, such as secondary anterior instability, avascular necrosis of the glenoid surface, and migration or resorption of the graft, have been reported in the literature.^15^^,^^16^^,^^20^ Lacheta et al.^15^ examined open posterior glenoid osteotomy with autologous bone grafting from the scapular spine. They reported 33% of nondisplaced glenoid neck fractures in 12 shoulders, with 91.7% achieving final stability.

Patients with atraumatic PSI in association with increased glenoid retroversion experienced inferior clinical outcomes. There was a trend toward a lower rate of return to sport compared with patients with traumatic PSI, suggesting that treating these patients may pose more significant challenges.^22^ Many authors argue that relying solely on soft tissue procedures is insufficient for addressing symptomatic PSI. Instead, they advocate for bone-block procedures and glenoid osteotomies, which typically result in better outcomes for patients with this condition.^11^^,^^13^^,^^20^

In a recent systematic review, Sardar et al.^23^ found that glenoid version was corrected by an average of 10° with a subsequent improvement in normal range of motion. Moreover, the overall function showed improvement as assessed by various scoring systems, including Constant-Murley, Rowe, Oxford instability, Japan Shoulder Society Shoulder Instability Scoring, and mean shoulder value. Additionally, they found a high complication rate (34%), including persistent posterior instability in 20% of patients and intraoperative iatrogenic glenoid neck and/or acromion fractures in 4% of cases. However, the revision rate was low, standing at 0.6%. In this review, 40% of the patients had osteoarthritis before surgery, and 6% of shoulders had degenerative changes postoperatively, and they concluded that this procedure should be performed on patients without or with mild osteoarthritis to reduce the high risk of complications. They recommend that qualified shoulder surgeons should perform the glenoid osteotomy.^23^ This recommendation highlights the complexity and potential risks involved in the procedure.

## Conclusions

The surgical arthroscopic posterior glenoid osteotomy technique is a reproducible, safe, and feasible option for treating recurrent posterior shoulder instability in patients with excessive glenoid retroversion. However, careful patient selection and experienced surgeons are better equipped to handle the challenges, ensure accurate execution, and reduce the likelihood of complications, ultimately leading to better patient outcomes.

## Supplementary Data

ICMJE author disclosure forms

Video 1 The patient is positioned in the lateral decubitus position with 30° posterior obliquity aligning the glenoid parallel to the floor, and the arm is placed in a lateral shoulder distraction system. The posterior scapula bone graft is harvested, and posterior, anterior, anterosuperolateral, and accessory posteroinferior portals are created. A posterior release of the capsulolabral complex is performed using a radiofrequency device and soft tissue elevator from the 6-o’clock position until behind the long head of the biceps tendon insertion at the 11-o’clock position. Care is taken to perform a full capsular release to ensure complete exposure and visibility of the posterior scapula bone. A percutaneous suture-passing technique is used to grasp and maintain the capsulolabral complex in a lateral position relative to the glenoid rim and create a large posterior articular pouch. A switching stick is inserted into the joint to establish the correct level of the posteroinferior working osteotomy portal, positioning it at the midpoint between the superior and inferior posterior glenoid rim. An open metallic cannula is then introduced over the switching stick. The switching stick is then removed, and the hook of the parallel drill guide is inserted and placed on the anterior glenoid rim. A split of the infraspinatus muscle is performed, and the drill guide sleeve is introduced after choosing a 7-mm glenoid rim offset. The guide is positioned carefully, ensuring contact with the glenoid rim both anteriorly and posteriorly. Two k-wires are drilled 1 cm apart and across the scapula, until contact is made with the anterior cortex. The drill guide is then removed, leaving the k-wires in place. The k-wires are then exposed posteriorly and a forked retractor with rails is introduced beneath the k-wires until contact is made with the posterior glenoid. An osteotome with depth marks is carefully introduced between the k-wires and the forked retractor until posterior bone contact is visualized. The forked retractor is then partially removed, and the osteotome is impacted into the bone until the 15-mm laser mark is reached. The osteotome is then retracted from the bone, and the orientation of the cutting tip is aligned inferiorly before impacting again to the desired 15-mm mark. Once again, retract the osteotome and change the orientation of the cutting tip superiorly to complete the glenoid osteotomy. The forked retractor with rails is introduced with the osteotome, and an open wedge osteotomy is performed carefully under direct visualization. The posterior scapular graft is attached to the bone graft inserter using 2 small k-wires. The graft is then inserted between the retractors and carefully impacted into the previously created posterior glenoid opening osteotomy. The k-wires are removed, and the graft is impacted until securely placed. The capsulolabral complex is then reattached to the edge of the posterior glenoid rim using 3 to 4 knotless 1.8-mm FiberTak soft anchors. To improve graft stability, at least 2 of the anchors must pass through the graft and osteotomy to reach the medial body of the scapula.
